# Poor-Law Infirmaries and Voluntary Hospitals

**Published:** 1913-02-22

**Authors:** C. Thackray Parsons

**Affiliations:** Medical Superintendent, Fulham Infirmary, Hammersmith


### POOR-LAW INFIRMARIES AND VOLUNTARY HOSPITALS.

By C. THACKRAY PARSONS, M.D.Lond., Medical Superintendent, Fulham Infirmary, Hammersmith.

The publication in The Hospital of Mr. A. P. Hughes Gibb's paper on co-operation in the treatment of the sick between Poor-Law infirmaries and voluntary hospitals raises once again a matter of the greatest interest to those concerned in the administration of these two classes of institutions. The suggestion he makes that there should be a system of co-operation by which all acute cases requiring institutional treatment should be treated in the voluntary hospitals and all chronic cases in Poor-Law infirmaries is one that has often been made before, and one that seems to have a peculiar attraction for many persons concerned in the general administration of the voluntary hospitals. Mr. Gibb states very ably all that can be said in favour of the scheme he advocates. I propose to criticise briefly the reasons he gives for its adoption, and then to state the disadvantages—disadvantages which appear to me to be so serious as to vitiate entirely the whole of his arguments.

Mr. Gibb gives three reasons in support of his proposition. He asserts (1) that the obscure case in a Poor-Law infirmary does not get the immense advantage of specialist diagnosis and treatment to be obtained in a voluntary hospital; (2) the valuable opportunities which such cases offer for the advancement of medical knowledge are lost; and (3) that in Poor-Law infirmaries there is a great mass of clinical material which is needed for medical education. It will be seen at once that even if one grants these three premises the conclusion drawn that all acute cases should be transferred from the infirmary to a voluntary hospital is a fine example of fallacious reasoning. The majority of acute cases are not obscure, and many obscure cases are chronic; not all voluntary hospitals are medical schools—the majority, indeed, are not—and the clinical material which hospitals lack, in the opinion of authorities on medical education, is the chronic case. The only inferences Mr. Gibb is entitled to draw from his premises are that all obscure cases, whether acute or chronic, should go to a voluntary hospital which is fully equipped with specialist departments, and that medical schools should be enabled to make use of the patients in Poor-Law infirmaries, especially the chronic cases, for clinical instruction. With these two conclusions in the present state of things I have very little to quarrel. As for the first it is obvious that the small medical staff of a Poor-Law infirmary cannot specialise in all branches of medicine and surgery, and the medical superintendent should be able to refer those cases his staff cannot deal with properly to appropriate special departments. As for the second there can be no question that the Poor-Law infirmaries should take their share in medical education. The medical staff of the infirmaries should be empowered to give clinical instruction to students sent by the medical schools on those cases, chiefly of a chronic, infectious, or contagious character, which are not usually admitted to or retained in a voluntary hospital.

Having dealt thus with the arguments Mr. Gibb brings forth in favour of the scheme—and I do not know that any others have ever been suggested—it remains to point out the disadvantages. They may be summarised under four heads: (1) The want of accommodation in the voluntary hospitals; (2) the disadvantages to the patients; (3) the injustice to the hospital medical staffs; and (4) the effects on the infirmary medical and nursing staffs.

### The Want of Accommodation in the Voluntary Hospitals.

As Mr. Gibb points out, the number of beds in the London infirmaries is nearly double the number in the voluntary hospitals. Moreover, of recent years the number of beds in the London infirmaries has increased (from 16,300 in 1906 to 18,248 in 1911), whereas the number of available beds in the London hospitals has remained almost stationary (9,538 in 1908 and 9,888 in 1910). Now, I do not know of any published figures showing the number of acute cases in the London infirmaries, but in 1911 I went through all the cases in the Fulham Infirmary on a given date and found that 51 per cent. of them would be classified as acute. Assuming much the same proportion holds in the other infirmaries, the number of acute cases in the London infirmaries would practically fill all the beds in the London hospitals. It would, as a matter of fact, completely swamp them, for included in the 9,888 beds of the London hospitals are 3,471 beds belonging to special hospitals, which would be unable to admit the ordinary class of acute case. As a matter of fact, most of the voluntary hospitals in London are unable to admit all the acute cases which seek admission to them as it is, and this notwithstanding the fact that they admit chronic cases only under exceptional circumstances, and transfer home or to Poor-Law infirmaries all cases which do not improve quickly under treatment. I have known cases of appendicitis, osteomyelitis, intestinal obstruction, pneumonia, fractured legs, concussion, etc., sent on to the nearest infirmary because all the beds in the hospital were full.

### The Disadvantages to the Patients.

Anyone who examines the positions of the voluntary hospitals on a map of London will see at once that their distribution is such as to render them most inconvenient to act as the sole hospitals for all acute cases. There are large areas in which no voluntary hospitals exist, and in many parishes the patients would have to be conveyed several miles. An expensive system of motor-ambulances would have to be established to meet this difficulty, and even then the risk of conveying patients in pain and acutely ill such distances would not be negligible. Moreover, there would be the expense and difficulty the patients' friends would be put to in visiting them. The friends of the ordinary inmates of hospitals can usually meet this expense without difficulty, but such expenditure would be quite beyond the means of most of the Poor-Law infirmary patients' friends, and both the patients and their friends would feel very deeply the inability to visit which would result. Indeed, one often hears patients in a Poor-Law infirmary say they did not go to a voluntary hospital because they did not want to go so far from their friends. Again, paradoxical as the assertion may at first sight appear, some of the patients in Poor-Law infirmaries are too proud and independent to go to a voluntary hospital. Time after time patients have said to me, "My doctor wanted me to go to a hospital, but I have never contributed to a hospital, and I don't want their charity. I have been a ratepayer in the past, and I feel I am entitled to treatment here." Such a feeling is perfectly reasonable and worthy of respect.

### The Injustice to the Hospital Staffs.

One of the great difficulties in bringing the voluntary hospitals into any scheme for the State treatment of the sick poor lies in the fact that the medical staff is unpaid. The medical profession has never been niggardly in giving free service to charitable agencies supported by voluntary contributions. When, however, the State assumes the responsibility for the treatment of any portion of the sick, and provides the necessary funds by compulsory levies upon all, the aspect of the matter is fundamentally changed. The medical profession maintains rightly, and of necessity, if its members are not to die of starvation, that work done for the State must be paid by the State. If all the acute Poor-Law cases are to be maintained in the voluntary hospitals at the expense of the rates, then the hospital staffs must be paid for their services. The ratepayers would naturally object to contribute the large sums required for the maintenance of their patients unless they were fully represented on the board of management, and the day would not be very far distant when the voluntary hospital would become the municipal hospital, and would be swallowed up in the Poor-Law system.

### The Effects on the Infirmary Medical and Nursing Staffs.

Mr. Gibb says that the whole burden of medical education has been thrown upon the voluntary hospitals. By "medical education" Mr. Gibb no doubt means the education of the medical practitioner. Even in this limited sense of the words the statement is not quite true, for a number of medical men pass annually after qualification from the voluntary hospital to the Poor-Law infirmary, where as assistant medical officers they gain experience in the treatment of chronic cases and the habit of self-reliance in dealing with acute cases and emergencies. But there is another phase of medical education in the wider significance of the term, and that is the education of the trained nurse. In that work the Poor-Law infirmaries are now taking a most important, if not a leading, part, for they probably train more nurses annually than the hospitals. And the training they offer is a most valuable one, for it includes every variety of case—acute, chronic, and infectious, surgical and medical—which the nurse is likely to meet with in private. The high efficiency the medical and nursing staffs of the separate Poor-Law infirmary have attained is directly due to the extreme variety of the cases admitted. To remove the acute cases would be to destroy to a great extent the interest of the work, to remove one of the great incentives to the maintenance of the efficiency of the staff, and to render it impossible to train nurses properly. Reforms are needed in the Poor-Law infirmaries, as they are in the voluntary hospitals; but the line of advance must be, not in the direction of shedding responsibilities and reducing the infirmary to the position of a home for incurables, but in providing the staff and the means to deal directly with the increasing complexities of diagnosis and treatment.

